# LncRNA SNHG6 functions as a ceRNA to regulate neuronal cell apoptosis by modulating miR‐181c‐5p/BIM signalling in ischaemic stroke

**DOI:** 10.1111/jcmm.14480

**Published:** 2019-07-23

**Authors:** Xi'an Zhang, Zhanhui Liu, Qing Shu, Shanqi Yuan, Zhiguo Xing, Jinning Song

**Affiliations:** ^1^ Department of Neurosurgery The First Affiliated Hospital of Xi'an Jiaotong University Xi'an China; ^2^ Department of Neurosurgery The Ninth Hospital of Xi'an Xi'an China; ^3^ Department of Pharmacy The Ninth Hospital of Xi'an Xi'an China

**Keywords:** BIM, competing endogenous RNA, ischaemic stroke, miR‐181c‐5p, SNHG6

## Abstract

Long non‐coding RNAs (lncRNAs) play important roles in the pathogenesis of brain and neurodegenerative disorders. As far as we know, the functions and potential mechanisms of small nucleolar RNA host gene 6 (SNHG6) in ischaemic stroke have not been explored. This study aimed to examine the functional role of SNHG6 in the ischaemic stroke. Middle cerebral artery occlusion (MCAO) in mice and the oxygen glucose deprivation (OGD)‐induced injury in neuronal cells were applied to mimic ischaemic stroke. TTC staining, quantitative real‐time PCR, cell apoptosis assay, caspase‐3 activity assay, Western blot, RNA immunoprecipitation and luciferase reporter assay were performed to evaluate the function and possible mechanisms of SNHG6 in the pathogenesis of ischaemic stroke. The results show that SNHG6 expression was significantly increased both OGD‐induced neuronal cells and MCAO model mice. In vitro results showed that inhibition of SNHG6 increased cell viability, inhibited cell apoptosis and caspase‐3 activity in OGD‐induced neuronal cells. Consistently, knockdown of SNHG6 reduced brain infarct size and improved neurological scores in the MCAO mice. Mechanistic study further revealed that SNHG6 functioned as a competing endogenous RNA (ceRNA) for miR‐181c‐5p, which in turn repressed its downstream target of Bcl‐2 interacting mediator of cell death (BIM) and inhibiting cell apoptosis. This study revealed a novel function of SNHG6 in the modulating neuronal apoptosis in the ischaemic stroke model, and the role of SNHG6 in the regulating of neuronal apoptosis was at least partly via targeting miR‐181c‐5p/BIM signalling pathway.

## 1 INTRODUCTION

Stroke is the second most common cause of death and major cause of disability worldwide. Because of the ageing population, the burden will increase greatly during the next decades, especially in developing countries.^1^ About 80% of all strokes are ischaemic stroke, and ischaemic stroke induces decreased brain blood flow, and triggers cascading events including inflammatory reaction, hypoxia, neurotoxicity of excitatory amino acids and cell apoptosis that may cause further cerebrovascular injuries and neuronal damage.^2^ The most prominent pathological change in cerebral ischaemia is the neuronal death, which leads to the irreversible loss of brain function.^3^ Growing evidence has suggested that neurons in the ischaemic penumbra or peri‐infarct region may mostly undergo apoptosis so that they are potentially recoverable for some time after the onset of stroke.^4^ Thus, it is important for us to elucidate the mechanisms underlying neuronal apoptosis, which may help us discover novel therapeutic approach in alleviating brain injury after ischaemic stroke.

Long non‐coding RNAs (lncRNAs) are defined as a class of transcripts longer than 200 nucleotides without protein‐coding potential.^5^ Recently, accumulating studies have revealed that a number of lncRNAs are not transcriptional noise but have important functions including exerting enhancer‐like activities, modulating transcription factor activity, recruiting histone modification complexes, co‐activating with neighbouring genes, and acting as transcriptional enhancers.^6^ A growing number of studies have demonstrated the aberrant expression of lncRNAs in various brain and neurodegenerative disorders. The lncRNA, small nucleolar RNA host gene 6 (SNHG6) is a housekeeping gene of the 5′–terminal oligopyrimidine tract family and associated with ribosomes. Currently, lncRNAs are found to exhibit differential functions in various human diseases, such as ischaemic stroke, hepatocellular carcinoma and breast cancer, and demonstrate the ability to affect cell transformation, metastasis and apoptosis.^7^, ^8^, ^9^ Previous study has revealed that SNHG6 promoted oesophageal squamous cell carcinoma cell proliferation and inhibited cell apoptosis.^10^ However, the functions and biological mechanisms of SNHG6 in the neuronal cells under ischaemic stroke condition have not yet been explored.

LncRNAs may functionally interact with a broad range of microRNA (miRNA) molecules through competitively binding with miRNAs, which results in miRNA degradation. This interaction have been discovered in various biological processes of human diseases such as ischaemic stroke and neurodegenerative diseases.^11^, ^12^

Bcl‐2‐interacting mediator of cell death (BIM) belongs to the Bcl‐2 family. Bcl‐2 can be activated by various pro‐apoptotic stimuli and promote cell death in a manner dependent on Bax and Bak.^13^ The role of BIM in ischaemic stroke has been elucidated in several studies. Meller et al, showed that rapid degradation of BIM mediated short‐term ischaemic tolerance in cultured neurons 14; Arumugam et al, found that gamma‐secretase‐mediated notch signalling induced neuronal cell death via NF‐κB‐BIM pathway in ischaemic stroke.^15^ In addition, studies also showed that the c‐Jun N‐terminal protein kinase signalling pathway mediated neurol apoptosis via interacting with BIM in focal cerebral ischaemia.^16^

In this study, we assessed the differential expression of SNHG6 in a mice middle cerebral artery occlusion (MCAO) model and oxygen glucose deprivation (OGD)‐induced cortical neuronal cells. The in vitro functional assays were performed to elucidate the underlying molecular mechanisms of SNHG6 in the pathogenesis of ischaemic stroke.

## 2 MATERIALS AND METHODS

### 2.1 In vivo mice MCAO model

Male C57BL/6 mice (6‐8 weeks, 21‐23 g) were purchased from Laboratory Animal Center, Xi'an Jiaotong University Health Science Center. Animals were cared for at least 1 week before experiments under the standard laboratory animal conditions (25°C, 12 hours light/dark cycle, 50%‐70% humidity) with free access to food and water. Experiments were carried out in accordance with Guidelines for the Care and Use of Laboratory Animals and approved by The First Affiliated Hospital of Xi'an Jiaotong University.

MCAO surgery was used to induce focal cerebral ischaemia in mice as reported previously.^17^ Mice were fasted 12 hours before surgery. Briefly, mice were anaesthetized by 3% chloral hydrate (300 mg/kg, ip.) and then right common carotid artery, internal carotid artery and external carotid artery were exposed. The nylon monofilament was used to occlude the middle cerebral artery. After 90 minutes of MCAO, reperfusion was accomplished by withdrawing the filament.

### 2.2 Determination of neurological deficit

The neurological deficits were evaluated after 24 hours of reperfusion. The neurological deficits in the MCAO mice were determined using 10‐score system as reported previously.^18^ The mice were eliminated from the experimental group with a neurobehavioural score <2.

### 2.3 Determination of infarct volume

Brain infarct volume was determined to assess brain injury. The mice were killed, brains were removed quickly. The brain samples were frozen at −20°C and cut into 1 mm coronal sections. The sections were incubated in 1% 2, 3, 5‐triphenyl‐tetrazolium chloride (TTC; Sigma, St. Louis, USA) and fixed with 4% paraformaldehyde overnight to assess the size of infarction.^19^

### 2.4 Primary mice cortical neuron culture

Primary mice cortical neuron culture was performed with a previously described method.^20^, ^21^ The cortices were obtained from embryos of pregnant mice under sterile conditions. The cortices digested with trypsin‐EDTA (1:1, 0.125%), then dissociated in minimum essential medium (MEM; Thermo Fisher Scientific, Waltham, USA) containing 10% foetal bovine serum (FBS; Thermo Fisher Scientific). After filtration, the cortical tissues were centrifuged at 1000 rpm for 5 minutes, and then resuspended in MEM with 10% FBS. Cortical neuron cells were plated into poly‐l‐lysine (10 mg/mL)‐coated plates. Cells were grown in Neurobasal Medium (NBM) with 3% B27 (Sigma). Cultures were maintained for 8 days before treatment and the medium was changed every 3 days.

### 2.5 In vitro OGD model

The primary cortical neurons were washed with warm glucose‐free DMEM (Thermo Fisher Scientific) and were introduced into an incubator containing 95% N_2_ and 5% CO_2_ at 37°C for 3 hours to establish OGD model. Then the cells were returned to high‐glucose DMEM under standard conditions with 95% O_2_ and 5% CO_2_.^22^, ^23^

### 2.6 Cell viability

The cell viability of cortical neurons was assessed using the 3‐(4,5‐dimethylthiazol‐2‐yl)‐2,5‐diphenyltetrazolium bromide (MTT; Sigma) assay.^24^ The treated cells were incubated with 50 μL of MTT solution (5 mg/mL) for 4 hours. After incubation, the supernatant was removed and 150 μL dimethyl sulfoxide was added. Then the optical density (OD) value at 490 nm was measured by microplate reader (Bio‐Tek, Winooski, USA).

### 2.7 Apoptosis assay

Apoptosis assay was performed according to previously published methods.^20^ Primary cortical neurons were collected from each group after different treatments. Cell apoptosis was detected by using an Annexin V‐fluorescein isothiocyanate propidium iodide (FITC/PI) apoptosis detection kit (Thermo Fisher Scientific) according to the manufacturer's protocol. Cells were harvested and washed with 1xphosphate buffered saline for twice. Cells were then incubated with Annexin V_FTIC and PI at room temperature for 15 minutes in the dark. The cell apoptosis was analysed by a FACScan flow cytometer (BD Biosciences, Franklin Lakes, USA).

### 2.8 Caspase‐3 activity assay

The cortical neurons were incubated in 6‐well plates after different treatments and the activity of caspase‐3 was measured using Caspase‐3 Assay kit (Abcam, Cambridge, UK) according to the manufacturer's instruction.

### 2.9 In vivo delivery of siRNAs into lateral ventricle of the mice

The in vivo administration of SNHG6 siRNA and scrambled siRNA were performed as described previously.^25^ Briefly, the anaesthetized animals were placed in a stereotaxic apparatus (David Kopf Instrument, Tujunga, USA) for intracerebroventricular (ICV) injection into the left ventricle. SNHG6 siRNA and control siRNA were carefully diluted with equal volumes of transfection reagent siPORT NeoFX (Invitrogen, Carlsbad, CA, USA) according to the guidelines. SNHG6 siRNA or control siRNA was injected into the animal brain (2 µL injection volume) using a Hamilton microsyringe at 1 day before MCAO surgery.

### 2.10 In vitro transfection

SNHG6 and Bcl‐2 interacting mediator of cell death (BIM) cDNA were amplified and subcloned into the KpnI and BamHI sites of pcDNA3.1 (GenePharma, Shanghai, China) to construct the pcDNA3.1‐SNHG6 and pcDNA3.1‐BIM vector, respectively. SNHG6 siRNA (si‐SNHG6), scramble negative control siRNA (si‐NC), miR‐181c‐5p mimic, miR‐181c‐5p control mimic(miR‐NC), miR‐181c‐5p inhibitor and miR‐181c‐5p inhibitor control were designed and synthesized by RiboBio Company (Guangzhou, China), and the transfection of these oligo nucleotides into primary cortical neurons was performed by using Lipofectamine 2000 reagent (Invitrogen) according to the manufacturer's instruction. At 24 hours post‐transfection, cells were processed for further in vitro assays.

### 2.11 Quantitative real‐time PCR (qRT‐PCR)

The total RNA and miRNAs from ischaemic brain tissues and cultured cells were extracted using the TRIzol reagent (Invitrogen) and miRNeasy Mini Kit (Invitrogen), respectively. For mRNA analysis, cDNA was synthesized by using M‐MLV reverse transcriptase (Invitrogen) and reverse transcription primers Oligo (dT). qRT‐PCR was then performed with SYBR Green Real‐Time PCR Master Mix (Thermo Fisher Scientific, Waltham, USA) on a 7900HT Fast Real‐Time PCR machine (Applied Biosystems, Foster City, CA, USA). For miRNA analysis, cDNA was obtained using the TaqMan MicroRNA Reverse Transcription Kit (Thermo Fisher Scientific) and qRT‐PCR was performed with TaqMan miRNA assay kit on a 7900HT Fast Real‐Time PCR machine (Applied Biosystems). The comparative Ct value method was used to determine the fold changes, and U6 and glyceraldehyde 3‐phosphate dehydrogenase were used as internal controls for miR‐181c‐5p and other genes, respectively.

### 2.12 Western blot

Total proteins were extracted from cells or tissues with radioimmunoprecipitation assay buffer on ice and the concentration of total protein was assessed by BCA assay (Bio‐Rad, Hercules, USA). Equal amounts of total proteins (25 µg) were separated by sodium dodecyl sulphate‐polyacrylamide gel electrophoresis and transferred to nitrocellulose membranes. Blocking was performed by incubating with 5% non‐fat milk for 1 hour at 37°C. After being washed three times with tris‐buffered saline with Tween 20, the membrane was incubated with primary antibodies including anti‐β‐actin (1:1000 dilution; Abcam), anti‐BIM (1:500 dilution; Abcam) overnight at 4°C. After overnight incubation, the membranes were further incubated with respective secondary antibodies at a dilution ratio of 1:2000 conjugated with horseradish peroxidase for 1 hour at room temperature. The Western blot bands were visualized by enhanced chemiluminescence kit (Thermo Fisher Scientific). The relative BIM protein levels were normalized to β‐actin.

### 2.13 Luciferase reporter assay

To construct luciferase reporter vectors, SNHG6 fragments containing the predicted miR‐181c‐5p binding site (both wild‐type and mutant) were amplified and cloned into the pGL3 luciferase vector (Promega, Madison, USA) and was named as SNHG6‐WT and SNHG6‐MUT, respectively. Cells were co‐transfected with SNHG6‐WT (or SNHG6‐MUT) and miR‐181c‐5p mimic (or miR‐NC) or inhibitor (or inhibitor‐NC) using Lipofectamine 2000 reagent (Invitrogen). At 48 hours post‐transfection, cells were harvested and luciferase activity was analysed with the dual luciferase reporter assay system (Promega) according to the manufacturer's instruction. Similarly, pGL3 luciferase reporter vector was used to construct wild‐type pGL3‐miR‐181c‐5p 3′ untranslated region (3′UTR; miR‐181c‐5p‐WT) or mutated pGL3‐miR‐181c‐5p 3′UTR (miR‐181c‐5p‐MUT) vectors. At 48 hours post‐transfection, the cells were harvested and luciferase activity was analysed with the dual luciferase reporter assay system (Promega).

### 2.14 RNA immunoprecipitation (RIP) assay

The physical interaction between SNHG6 and miR‐181c‐5p was evaluated using the Magna RIP^™^ RNA Binding Protein Immunoprecipitation Kit (Millipore, Burlington, USA) according to the manufacturer's instructions. Briefly, cells after miRNAs transfections were lysed in the RIP lysis buffer, followed by incubating the lysates with RIP buffer containing magnetic beads conjugated with negative control IgG or anti‐Argonaute2 (Ago2) antibody according to the manufacturer's protocol. The precipitation of the RNA was purified and processed for further qRT‐PCR analysis.

### 2.15 Statistical analysis

The experimental data are expressed as mean ± standard deviation (SD). Statistical analysis was performed with the t‐test or one‐way analysis of variance (ANOVA) followed by Bonferroni's multiple comparison test. *P* < 0.05 was considered to statistically significant. All statistical figures were plotted by using GraphPad Prism Version 5.0 (GraphPad Software, La Jolla, USA). Statistical analysis was analysed by SPSS software 15.0 (IBM, Armonk, USA).

## 3 RESULTS

### 3.1 SNHG6 expression was elevated after MCAO in mice and after OGD in cultured cortical neurons

**Figure 1:**
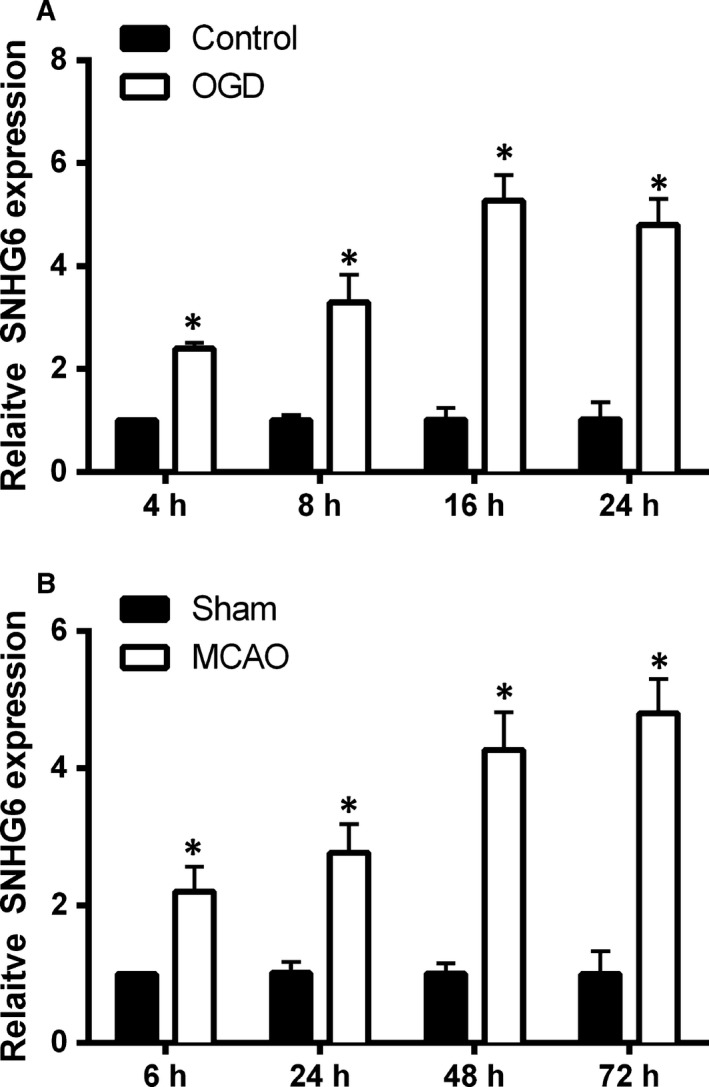
The expression of SNHG6 in cerebral ischaemia in vitro and in vivo models. A, qRT‐PCR analysis for expression of SNHG6 in the cortical neurons at 4, 8, 16 and 24 h after OGD treatment (N = 3). B, qRT‐PCR analysis for expression of SNHG6 in mice ischaemic brain tissues at 6, 24, 48 and 72 h after MCAO (N = 6). Data were shown as mean ± SD *P < 0.05

We first determined changes of SNHG6 expression in cultured cortical neurons after OGD at time points of 4, 8, 16 and 24 hours. Compared with control group, expression levels of SNHG6 in OGD group were significantly increased at 4, 8, 16 and 24 hours (Figure 1A).

The changes of SNHG6 expression after focal ischaemic stroke were also determined in mice that were subjected to MCAO for 90 minutes followed by reperfusion. SNHG6 expression in different groups was assessed at 6, 24, 48 and 72 hours after MCAO. The qRT‐PCR results show significantly increased levels of SNHG6 expression in the MCAO group at 6, 24, 48 and 72 hours after MCAO when compared to sham‐control group (Figure 1B), and the up‐regulation of SNHG6 induced by MCAO was displayed in a time‐dependent manner (Figure 1B).

### 3.2 Knockdown of SNHG6 inhibits OGD‐induced neuronal cells injury

**Figure 2:**
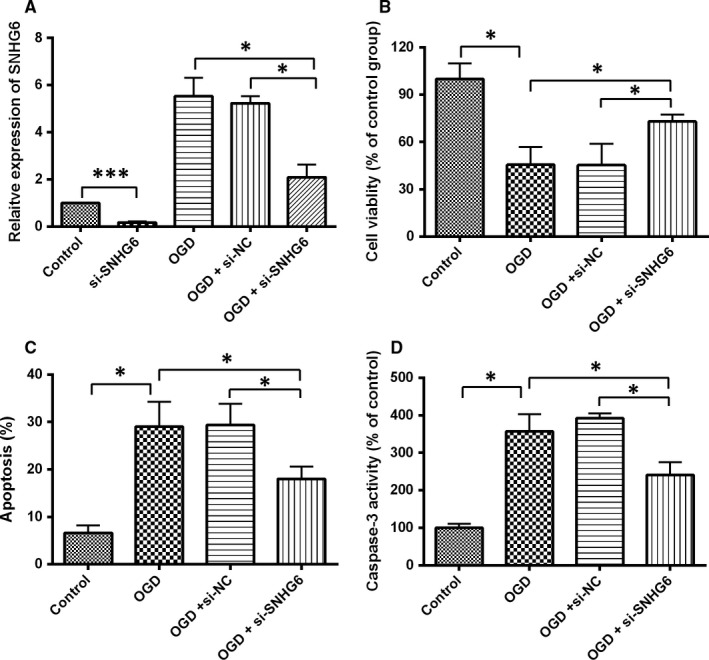
Knockdown of SNHG6 inhibited OGD‐induced neuronal apoptosis. A, qRT‐PCR analysis for expression of SNHG6 in cultured cortical neuron cells in each group (N = 3). B, Cell viability was determined by MTT assay in cortical neurons received different treatments (N = 3). C, Cell apoptosis was evaluated by flow cytometry in cortical neurons received different treatments (N = 3). D, Caspase‐3 activity was determined by caspase‐3 activity assay in cortical neurons received different treatments (N = 3). Data were shown as mean ± SD *P < 0.05, ***P < 0.001

To determine the role of SNHG6 in ischaemic stroke, we used OGD‐induced cortical neurons to mimic ischaemic stroke in vitro. As indicated in Figure 2A, no significant difference was found in SNHG6 expression between the OGD + si‐NC group and OGD alone group. With the SNHG6 siRNA transfection, the SNHG6 expression in si‐SNHG6 group or in OGD + si‐SNHG6 group was decreased notably compared to their respective control groups. Silencing of SNHG6 promoted cell viability and decreased OGD‐induced neuronal apoptosis (Figure 2B,C). Moreover, compared with OGD group, down‐regulation of SNHG6 reduced caspase‐3 activity (Figure 2D). In general, these data indicated that silencing of SNHG6 inhibited OGD‐induced neuron cells injury.

### 3.3 Knockdown of SNHG6 alleviates cerebral infarction and reduces neurological score

**Figure 3:**
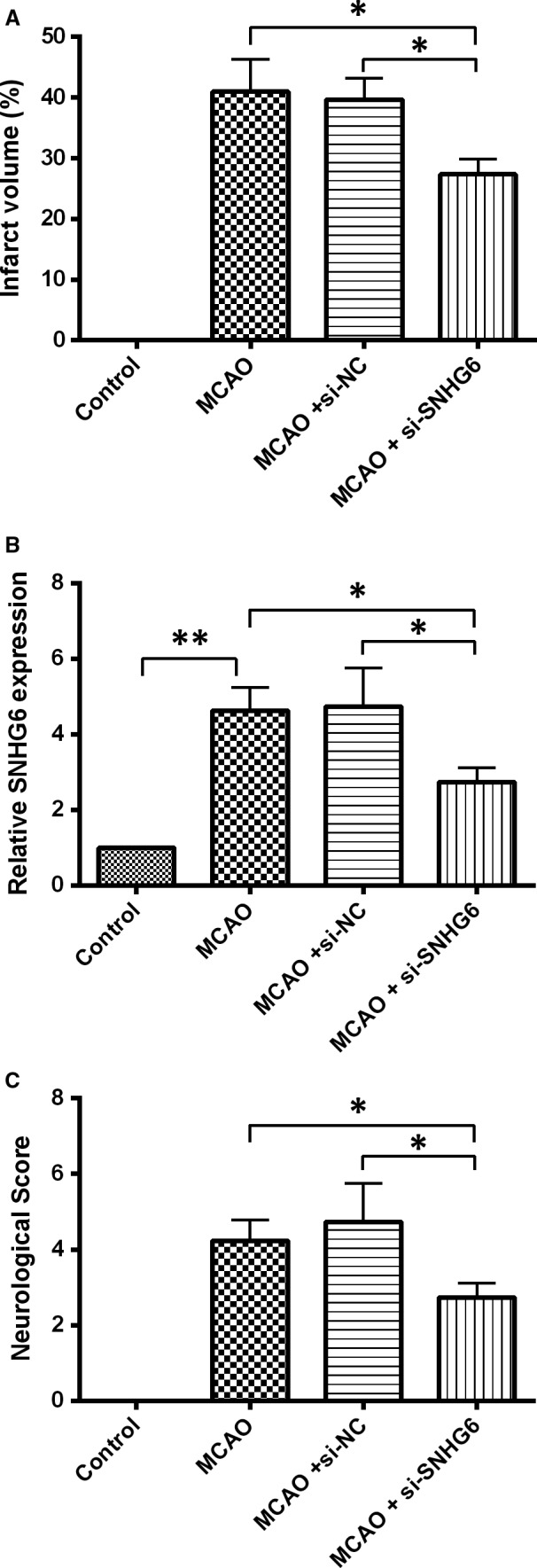
Knockdown of SNHG6 improved ischaemic stroke outcome in mice MCAO model. A, Representative TTC staining images of brain slices and quantification of brain infarct volume in mice with different treatments (N = 6). B, qRT‐PCR analysis for expression of SNHG6 in the brain tissues from the mice received different treatments (N = 6). C, The neurological deficit score data in the mice with different treatments (N = 6). Data were shown as mean ± SD *P < 0.05 and **P < 0.01

The infarct volume in the mice brain was detected by TTC staining. Viable brain tissue was stained with red colour and the infarct tissue remains white. As shown in Figure 3A,B, no infarct area was detected in control group, while in the MCAO group, the infarct volume was increased significantly. Knockdown of SNHG6 markedly alleviated cerebral infarction volume when compared to MCAO + siNC group (Figure 3A,B). In addition, the neurological score increased significantly in the MCAO group, and compared with the OGD + si‐NC group, mice in the SNHG6 siRNA group showed remarkably decreased neurological deficit (Figure 3C).

### 3.4 SNHG6 directly binds to miR‐181c‐5p and negatively regulates its expression

**Figure 4:**
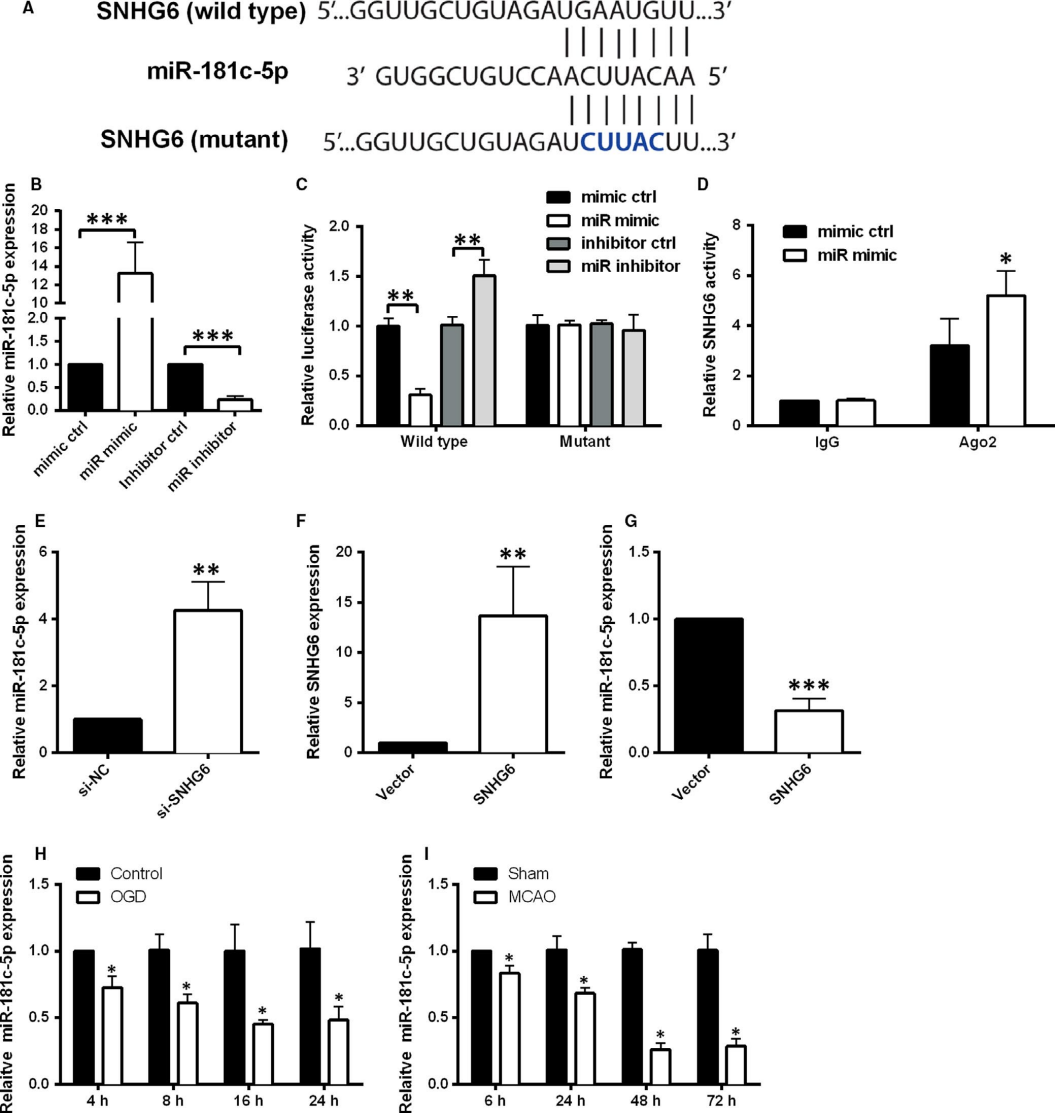
SNHG6 binds directly to miR‐181c‐5p and negatively regulates the expression of miR‐181c‐5p. A, The putative miR‐181c‐5p binding sites of the wild‐type and mutant sequence of SNHG6. B, qRT‐PCR analysis for expression of miR‐181c‐5p in cortical neurons transfected with miR‐181c‐5p mimic, mimic control, miR‐181c‐5p inhibitor or inhibitor control (N = 3). C, Luciferase activity was detected in cortical neurons co‐transfected with miR‐181c‐5p mimic (or mimic control) and reporter vector containing SNHG6 WT (or SNHG6‐MUT) (N = 3). D, RIP analysis for the interaction between SNHG6 and miR‐181c‐5p in cortical neurons with miR‐181c‐5p mimic or mimic control transfection. E, qRT‐PCR analysis for the expression of miR‐181c‐5p in cortical neurons transfected with si‐SNHG6 or si‐NC (N = 3). F, qRT‐PCR analysis for the expression of SNHG6 in cortical neurons transfected with pcDNA3.1 (Vector group) or pcDNA3.1‐SNHG6 (SNHG6 group). G, qRT‐PCR analysis for the expression of miR‐181c‐5p in cortical neurons transfected with pcDNA3.1 (Vector group) or pcDNA3.1‐SNHG6 (SNHG6 group). H, qRT‐PCR analysis for expression of miR‐181c‐5p in cortical neurons at 4, 8, 16 and 24 h after OGD treatment (N = 3). I, qRT‐PCR for expression of miR‐181c‐5p in ischaemic brain tissues at 6, 24, 48 and 72 h after MCAO (N = 6). Data were shown as mean ± SD *P < 0.05, **P < 0.01 and ***P < 0.001

Bioinformatics analysis was conducted using Diana tools and the results revealed that miR‐181c‐5p was one of the predicted targets (Figure 4A), and miR‐181c‐5p was selected for further study due to its regulatory role in cell apoptosis.^26^, ^27^, ^28^ Transfection with miR‐181c‐5p mimic significantly increased the expression level of miR‐181c‐5p compared with mimic control group, and transfection with miR‐181c‐5p inhibitor down‐regulated miR‐181c‐5p expression (Figure 4B). The luciferase assay data showed that miR‐181c‐5p mimic transfection apparently inhibited the luciferase activity of the reporter vector containing SNHG6‐WT, and miR‐181c‐5p inhibitor increased the luciferase activity; however, both miR‐181c‐5p mimic and inhibitor failed to influence the luciferase activity of reporter vector containing SNHG6‐MUT (Figure 4C). For the RIP analysis, we found that endogenous SNHG6 was significantly enriched in the cells with miR‐181c‐5p mimic transfection when compared to mimic ctrl transfection (Figure 4D). Knockdown of SNHG6 in the neurons notably increased miR‐181c‐5p expression (Figure 4E). Overexpression of SNHG6 in the neurons suppressed the expression of miR‐181c‐5p (Figure 4F,G). Furthermore, we assessed miR‐181c‐5p expression in cortical neurons after OGD at different time points, and the qRT‐PCR results showed that the expression level of miR‐181c‐5p in cortical neurons after OGD was significantly increased at 4, 8, 16 and 24 hours after treatment compared to control group (Figure 4H). We also determined changes of miR‐181c‐5p expression in brain tissues after MACO. Consistently, expression of miR‐181c‐5p in MCAO group was decreased evidently at 6, 24, 48 and 72 hours after surgery when compared to sham group (Figure 4I). These results indicated that SNHG6 directly bound to miR‐181c‐5p and negatively regulated the expression of miR‐181c‐5p.

### 3.5 miR‐181c‐5p targets the 3′UTR of BIM and negatively regulates the expression of BIM

**Figure 5:**
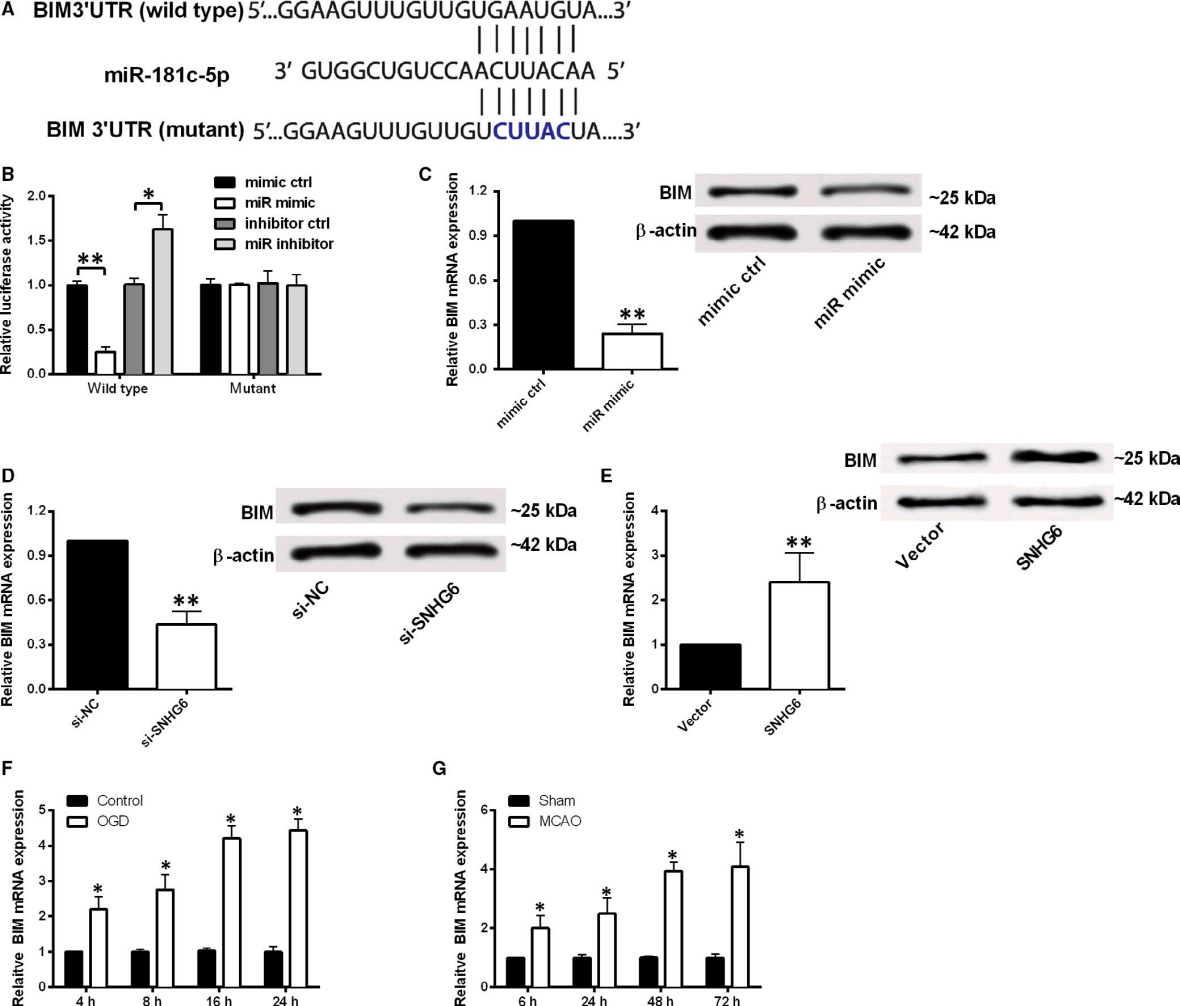
BIM is a target of miR‐181c‐5p and was negatively regulated by miR‐181c‐5p. A, The putative BIM binding site of the wild‐type and mutant sequence of miR‐181c‐5p. B, Luciferase activity was detected in cortical neurons co‐transfected with miR‐181c‐5p mimic (or mimic control) and reporter vector containing miR‐181c‐5p‐WT (or miR‐181c‐5p‐MUT) (N = 3). C, Relative mRNA and protein expression of BIM in cortical neurons transfected with miR‐181c‐5p mimic or mimic control. D, Relative expression of BIM mRNA and protein in cortical neurons transfected with si‐SNHG6 or si‐NC. E, Relative expression of BIM mRNA and protein in cortical neurons transfected with pcDNA3.1 (Vector group) or pcDNA3.1‐SNHG6 (SNHG6 group). (F) qRT‐PCR analysis for expression of BIM in at 4, 8, 16 and 24 h after OGD treatment (N = 3). (G) qRT‐PCR for expression of BIM in ischaemic brain tissues at 6, 24, 48 and 72 h after MCAO (N = 6). Data were shown as mean ± SD *P < 0.05 and **P < 0.01

Bioinformatics analysis was conducted by using Targetscan tool, which revealed that BIM was one of the predicted genes targeted by miR‐181c‐5p (Figure 5A). The luciferase assay data showed that miR‐181c‐5p mimic transfection apparently inhibited the luciferase activity of reporter vector containing miR‐181c‐5p‐WT, and miR‐181c‐5p inhibitor transfection increased the luciferase activity; however, both miR‐181c‐5p mimic and inhibitor failed to modulate the luciferase activity of reporter vector containing miR‐181c‐5p‐MUT (Figure 5B). Decreased BIM mRNA and protein expression levels were observed in neurons being transfected with miR‐181c‐5p mimic or si‐SNHG6 when compared to their respective negative controls (Figure 5C,D). Overexpression of SNHG6 also increased the mRNA and protein expression of BIM in cortical neurons (Figure 5E). In addition, we assessed BIM expression in cortical neuronal cells after OGD at different time points, and the qRT‐PCR results show BIM mRNA expression increased obviously in OGD group at 4, 8, 16 and 24 hours after treatment compared to control group (Figure 5F). We also determined changes in BIM mRNA expression in brain tissues after MCAO. Similarly, the mRNA expression level of BIM in MCAO group increased evidently at 6, 24, 48 and 72 hours after surgery when compared to sham group (Figure 5G).

### 3.6 SNHG6 functions as a competing endogenous (ceRNA) for miR‐181c‐5p to regulate BIM expression and promote cell apoptosis

**Figure 6:**
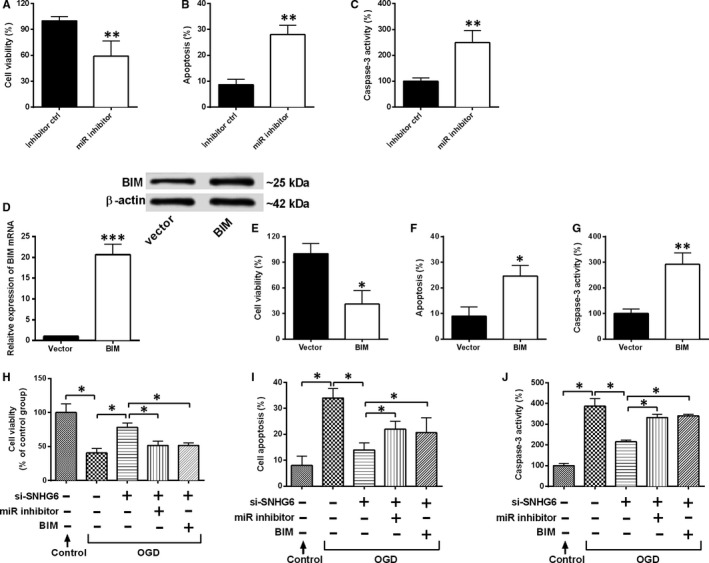
SNHG6 functions as a ceRNA for miR‐181c‐5p to regulate BIM expression. A, Cell viability, B, cell apoptosis and C, caspase‐3 activity were evaluated in cortical neurons transfected with miR‐181c‐5p inhibitor or inhibitor control (N = 3). D, Relative mRNA and protein expression levels of BIM in cortical neurons transfected with pcDNA3.1‐BIM or pcDNA3.1 (N = 3). E, Cell viability, F, cell apoptosis and G, caspase‐3 activity were evaluated in cortical neurons transfected with pcDNA3.1‐BIM or pcDNA3.1 (N = 3). H, Cell viability, I, cell apoptosis and J, caspase‐3 activity were measured in cortical neurons co‐transfected with different siRNAs, miRNAs or plasmids (N = 3). Data were shown as mean ± SD *P < 0.05, **P < 0.01 and ***P < 0.001

BIM is an apical signalling protein, which has been shown to directly activate Bax promoting engagement of programmed cell death mechanisms,^29^ and we further explored relationship between SNHG6 and BIM. Knockdown of miR‐181c‐5p suppressed cell viability, increased cell apoptosis and caspase‐3 activity (Figure 6A‐C). Then, we explored the role of BIM in cortical neurons. Increased mRNA and protein expression of BIM in the pcDNA3.1‐BIM group were observed when compared with vector group (Figure 6D). Decreased cell viability, enhanced neuron apoptosis and caspase‐3 activity were also found in pcDNA3.1‐BIM group when compared to vector control group (Figure 6E‐G).

Furthermore, we determined whether SNHG6 regulated neuron apoptosis through the activation of miR‐181c‐5p/BIM pathway. Knockdown of miR‐181c‐5 or overexpression of BIM attenuated the effects of SNHG6 down‐regulation on cell viability and cell apoptosis in the OGD‐treated neurons (Figure 6H‐J).

## 4 DISCUSSION

Stroke is defined as the clinical syndrome of rapid onset of focal or global cerebral deficit.^30^ In recent years, stroke has become the second leading cause of death and the most important cause of disability in many countries.^31^ In this study, we explored the role of SNHG6 in ischaemic stroke both in vitro and in vivo. Expression of SNHG6 significantly increased at different time points after MCAO in the mice and after OGD treatment in the cortical neurons. Knockdown of SNHG6 decreased the brain infarct volume and alleviated neurological deficit apparently. In addition, we found that silencing of SNHG6 increased cell viability, inhibited neuronal apoptosis and caspase‐3 activity. By further exploring the underlying molecular mechanisms, we demonstrated that SNHG6 regulated cell viability and cell apoptosis of cortical neurons through the modulation of miR‐181c‐5p/BIM signal pathway.

SNHG6 demonstrated a high degree of conservation and was ineffectively degraded by nonsense‐mediated mRNA decay (NMD), which indicated that it may have extra functions apart from the production of U87 RNA.^32^ Fan et al 10 indicated that SNHG6 is up‐regulated in oesophageal cancer, and inhibition of SNHG6 resulted in diminished cell growth and increased apoptosis. Another study shown that overexpression of SNHG6 functioned as a miRNA sponge and could promote cell proliferation and suppress cell apoptosis via modulating miR‐101‐3p and ZEB1 expression in gastric cancer.^33^ In our study, we for the first time provided substantial evidence to demonstrate that SNHG6 was apparently up‐regulated in mice MCAO brain tissues and OGD‐induced cortical neuron cells, and our results suggest that knockdown of SNHG6 may alleviated the ischaemic stroke in the mice likely via inhibiting the cell apoptosis.

In recent years, ceRNA has been proposed as a new regulatory mechanism in multiple pathological and physiological processes.^34^, ^35^ MiRNAs are a class of small non‐coding RNA molecules with ~23 nucleotides in length, and regulated its downstream gene expression via targeting the 3′UTR of the genes.^36^ In our study, we found that miR‐181c‐5p was a downstream target of SNHG6 and was negatively regulated by SNHG6. The miR‐181 family is evolutionarily conserved and highly expressed in the brain，and has been implicated for its important roles in the cell processes including apoptosis.^37^, ^38^ Our results further showed that knockdown of miR‐181c‐5p inhibited cell viability and increased cell apoptosis in cortical neurons and attenuated the effect of SNHG6 down‐regulation on OGD‐treated cortical neurons. Bioinformatics analysis showed that BIM was one potential target of miR‐181c‐5p. BIM, as a member of BH3 Subfamily, plays an important role in the initiation and regulation of apoptosis, and studies have found that BIM was found to be overexpressed in the ischaemic regions in the mice and rat MCAO model.^39^, ^40^ In vitro functional assays showed that miR‐181c‐5p could negatively regulate the expression of BIM, suggesting that miR‐181c‐5p plays an anti‐apoptotic role through its inhibitory effect on BIM. In this study, we have demonstrated that overexpression of BIM inhibited cell viability and increased cell apoptosis in cortical neurons and attenuated the effect of SNHG6 down‐regulation on OGD‐treated cortical neurons, indicating that pro‐apoptotic effects of SNHG6 by regulating miR‐181c‐5p/BIM signalling.

The present study also has some limitations. The expression of SNHG6 was only confirmed in the in vitro cell culture and in vivo animal models, and the role of SNHG6 from the clinical perspective should be determined in future studies. Based on the results from bioinformatics prediction, the downstream targets of SNHG6 were not limited to miR‐181c‐5p, and other predicted miRNAs may be investigated to consolidate the role of SNHG6 in ischaemic stroke. As SNHG6 affected the expression of BIM in the present study, whether SNHG6 also affected other apoptotic‐related proteins that interact with BIM should also be considered to further reveal the mechanistic role of SNHG6 in the regulation of cell apoptosis.

In conclusion, our study for the first time demonstrated that lncRNA SNHG6 functions as a ceRNA to regulate neuronal death by targeting miR‐181c‐5p/BIM signalling pathway in ischaemic stroke. This study may provide a better understanding of the functions and potential mechanisms of SNHG6 in the pathogenesis of ischaemic stroke. Further may be warranted to clarify the molecular mechanisms of the cross‐talk signalling pathway regulated by SNHG6 in the pathogenesis of ischaemic stroke.

## CONFLICT OF INTEREST

The authors declare that they have no conflict of interest.

## AUTHOR CONTRIBUTIONS

XZ and JS designed and wrote the manuscript, XZ, ZL and QS performed the experiments and SY and ZX contributed to the statistical analyses. All authors read and approved the final manuscript.
